# Targeted theranostic nanomedicine using targeted CT-imageable particles that release tebentafusp.

**DOI:** 10.1007/s11604-025-01782-w

**Published:** 2025-04-17

**Authors:** Satoshi Harada, Takahiro Sato, Kunihiro Yoshioka

**Affiliations:** 1 Department of Radiology, School of Medicine, Iwate Medical University, 1-1 1-Chome Idai-Dori, Yahaba, Shiwa 028-3694 Japan; 2 National Institutes for Quantum Science and Technology, Takasaki Ion Accelerators for Advanced Radiation Application, Foundational Quantum Technology Research Directorate, Takasaki Institute for Advanced Quantum Science, 1233 Watanuki, Takasaki, Japan

**Keywords:** Theranostics, CT-imageable nanoparticles, Radiotherapy, Tebentafusp, Micro-PIXE camera

## Abstract

**Purpose:**

A theranostic nanomedicine for CD3^+^ bispecific antibodies targeting glycoprotein-100 (GP-100) was tested in vivo using two radiation sessions. CT-imageable nanoparticles composed of hyaluronate-alginate and designed to release their contents upon radiation exposure were evaluated in a mouse model of B16-melanoma model in the left hind leg with pulmonary metastases.

**Materials and Methods:**

In session 1, IFN-γ was encapsulated during the Fe polymerization of hyaluronate-alginate nanoparticles. Nine hours after the intravenous injection of 1 × 10^10^ IFN-γ nanoparticles, enough to observe dose escalation of either 10 or 20 Gy was administered using 140 keV-X-ray to the primary and metastatic tumors. In session 2, tebentafusp was encapsulated using the same method as in session 1. Seventy-two hours after the intravenous injection of 1 × 10^10^ tebentafusp-loaded nanoparticles, radiation was administered under conditions identical to those in session 1.

**Results:**

In session 1, IFN-γ-loaded nanoparticles selectively accumulated in the primary tumor and pulmonary metastasis by passing through the coarse endothelium of tumor vasculature, which could be visualized using CT. IFN-γ nanoparticles continuously released IFN-γ, facilitating the formation of the HLA-A*02:01-GP100-complex. In session 2, the tebentafusp-loaded nanoparticles continuously released tebentafusp, leading to the formation of an immunological synapse consisting of HLA-A*02:01-GP100, tebentafusp, and CD3 on T cells. CD3^+^ T cells release perforin and granzymes, resulting in the cytolysis of the primary tumor and pulmonary metastasis. This effect was synergistic with that of radiation, resulting in Enhancement Factor (EF) more than 1.

**Conclusion:**

Theranostic nanomedicine demonstrated potential as a dual therapeutic and diagnostic strategy for targeting tumors and metastases, with synergistic effects observed when combined with radiation.

**Graphical abstract:**

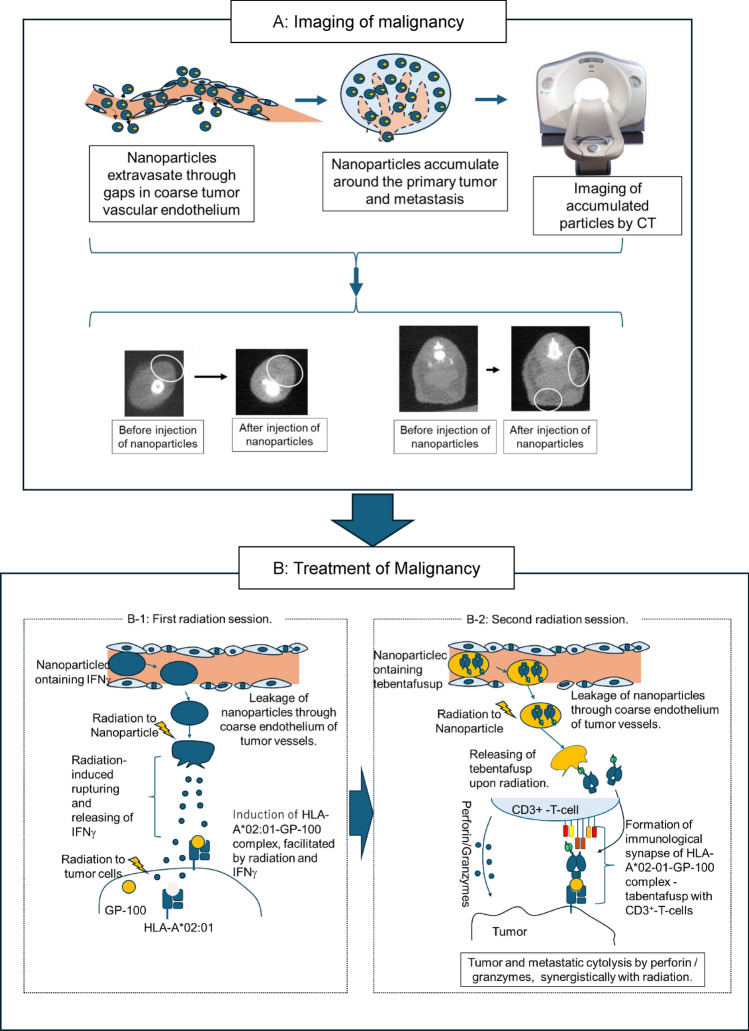

## Introduction

Radiation induces IFN-γ-mediated CD3^+^ T-cell infiltration [1], and enhances MHC class I and II expression, including HLA-A*02:01, which contains glycoprotein 100 (GP-100), a protein highly expressed in melanoma and upregulated by IFN-γ [2–6]. Tebentafusp, a bispecific antibody with high affinity for radiation-recruited CD3^+^ T cells and radiation-induced HLA-A*02:01-GP-100, forms an immunological synapse, leading to GP-100-HLA-A*02:01-Tebentafusp-CD3^+^ T cells. Radiation-recruited CD^3+^ T cells release granzymes and perforin, resulting in the death of the melanoma cells [4, 7, 8]. The enhanced antitumor effect observed in melanoma cells can be attributed to the combination of radiation and tebentafusp. Therefore, tebentafusup is considered to be an optimal immunotherapeutic agent to be used in combination with radiation. Previously, we developed computed tomography (CT)-detectable nanoparticles that released their contents upon irradiation for radiation-directed drug delivery [9–11].

This study tested a theranostic nanomedicine for CD3^+^ bispecific antibodies targeting glycoprotein-100 (GP-100) in vivo using two radiation sessions CT-imageable nanoparticles composed of hyaluronate-alginate and designed to release their contents (IFNγ or tebentafusp) upon radiation exposure were evaluated in a C57BL mouse model of B16-melanoma in the left hind legs with pulmonary metastases,which lead to better diagnosis and radio-immunotherapy of malignant melanoma in clinics.

## Materials and methods

### Overview

Our theranostic nanomedicine consisted of two components: A: Imaging of malignancy, followed by B: treatment of malignancy.

**Fig. 1:**
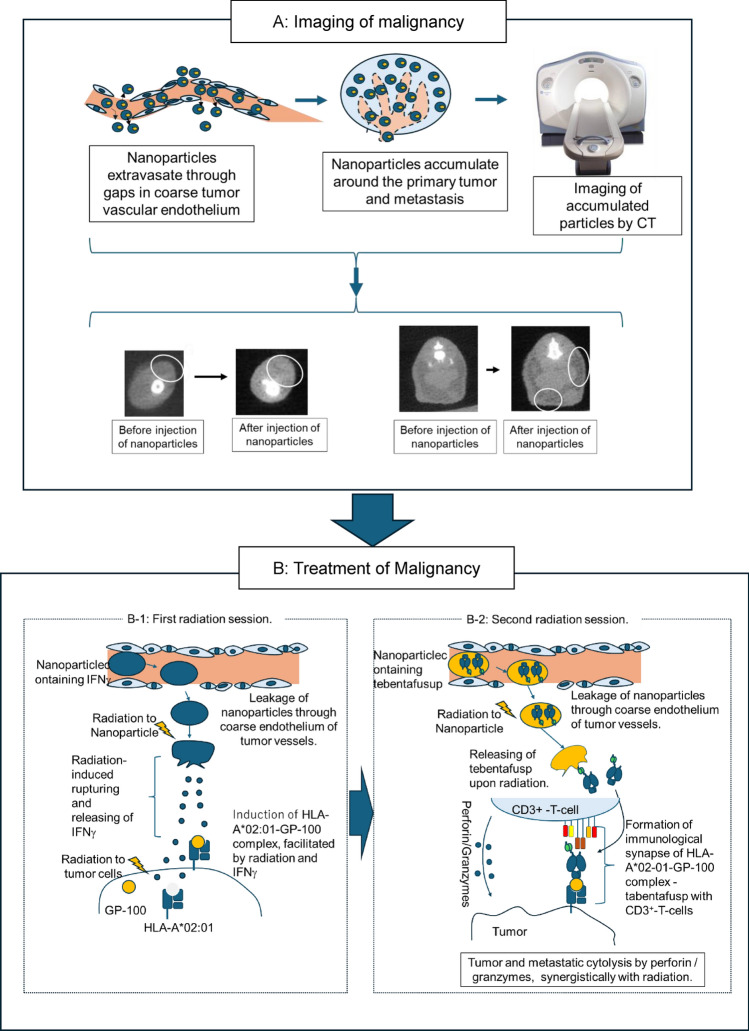
Graphical abstract. Two componenents of this theranostics. **A**: Imaging of malignancy using CT-detectable nanoparticles. **B**: Targeted immunotherapy using particles that release their contents upon irradiation, consisted of following two sub-components. B-1: induction of HLA-A*02–01-GP100 and B-2:formation of immunological synapse and releasing of perforin/granzymes from CD3^+^ T-cells

Regarding imaging of malignancy (Fig. 1A), CT-detectable nanoparticles containing IFN-γ were intravenously injected and accumulated around the tumor by exiting the blood vessel through the coarse vascular endothelial gap. The accumulated CT-detectable nanoparticles were imaged by CT and contributed to the detection of primary tumors and metastases.

**Fig. 2:**
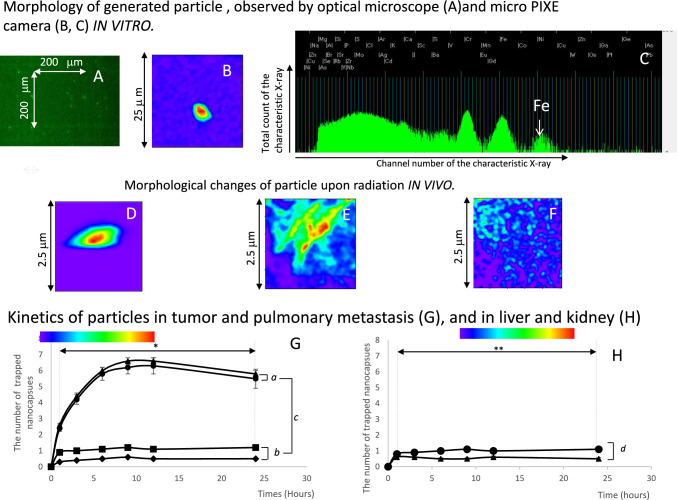
Morphology and kinetics of particles. **A**: The generated nanoparticles on the hemocytometer by optical microscope (400 ×). **B**: The generated nanoparticles observed through the micro-PIXE camera (25 × 25 μm). **C**: The spectrum of induced characteristic X-rays of particles (Total count of characteristic X-ray VS Channel number of characteristic X-ray).The spectrum of Fe used for particle imaging is indicated. **D**: Nanoparticles without irradiation (2.5 × 2.5 μm): Type I (unruptured). **E**: Irradiated Nanoparticles (2.5 × 2.5 μm): Type II (ruptured). **F**: Irradiated Nanoparticles: Type III (2.5 × 2.5 μm) (uptured). **G**: Kinetics of injected nanoparticles. ◆: Muscles, ■: Lung, ●: Primary tumor, ▲: pulmonary metastasis. *a, b:* No significant different in each observation time points;*c:* Significant different from 1 to 24 h after injection . **H**: Kinetics of particles in the liver and kidneys. ▲: Liver, ●: kidneys. *d:* No significant different from 1 to 24 h after injection**

Following imaging of malignancies (Fig. 1A), our theranostics progressed to the treatment phase (Fig. 1B), which consists of two steps: (1) induction of HLA*02–01-GP-100 on the tumor surface by a first radiation session (Fig. 1B-1); and (2) followed by targeting of tebentafusup by a second radiation session (Fig. 1B-2).

In the first radiation session (Fig B-1), the nanoparticles detected by CT imaging released IFNγ in response to first radiation. The released IFNγ cooperated with first radiation to induce HLA*02–01-GP-100 on the tumor surface.

Next, the Second radiation session is conducted (Fig. 1B-2). Nanoparticles containing tebentafusup were again injected intravenously and accumulated around the tumor, as seen in CT imaging and the first radiation session, and released tebentafusup in response to the second radiation. The released tebentafusup connects HLA*02–01-GP-100, which was induced on the tumor surface during the first radiation session (Fig. 1B-1), with CD3^+^ T-cells (Fig. 1B-2). Finally, the immunological synapse of HLA*02–01-GP-100-tebentafusup was formed, and Granzymes/Perforin was released from CD3^+^-T-cells, which attacked the malignant tumor (Fig. 1B-2).

### Preparation of nanoparticles

A 10 mL aqueous solution containing encapsulation chemicals (9-ng IFN-γ for the first radiation session, 200-ng tebentafusp for the second radiation session) was mixed with 0.2% (weight/volume) alginate and 0.1% hyaluronic acid. The mixture was sprayed into the vibrating solution of 0.3 mmol CaCl_2_ and 0.3 mmol FeCl_2_, using an ultrasound disintegrator (Branson Sonifier 150; Emerson, St. Louis, MO, USA) at 9 W output power. The polymerization of alginate-hyaluronate by FeCl_2_ and CaCl_2_ was completed within 5 min. The generated nanoparticles were purified using a 0.5 μm cellulose filter and re-suspended in 0.1 mol/L Tris (hydroxymethyl) aminomethane buffer (pH 7.4) [10, 11]. The particles were used in experiments within 6 h after generation at a concentration of 1X10 particles, floated in 1 ml of 0.1 mol/L Tris (hydroxymethyl) aminomethane buffer (pH 7.4) at 4 C in the dark. The stability of the particles was confirmed by observing no morphological changes by optical microscope and no release of contents by a micro PIXE camera (described in following Sect.  Discussion. Micro PIXE camera).

### Tumor models

B-16 melanoma cells were cultured in Eagle’s minimal essential medium supplemented with 10% fetal bovine serum and 1% penicillin–streptomycin. After trypsinization, 1 × 10^6^ viable cells were injected subcutaneously into the left hind leg of 6-week-old male C57BL/6 mice to generate a primary tumor, and 2 × 10^6^ viable cells were injected intravenously to induce lung metastasis. The primary tumor grew to an oval shape, measuring 8 mm, with 8–10 lung metastases observed at that time. Experiments with eight mice in each group followed the guidelines of the Animal Experiments of Iwate Medical University (No. H27-30),

### Sampling of primary tumor and lung metastases

The mice were euthanized using a CO_2_ chamber and their tumors, lungs, liver, and kidneys were excised. Each tissue sample was cut into two pieces: one was frozen at -80 °C for the micro-PIXE analysis to measure for IFN-γ, perforin, and granzymes, while the other was stored in Boulin’s fluid for immunohistochemical analysis of the HLA-A*02–01-GP-100 complex and CD3^+^ T cells infiltration.

### Micro-PIXE camera analysis and determination of nanoparticle rupturing upon radiation

Particle suspensions or 3 μm tissue sections were placed on a 1 μm Mylar film, dried under vacuum (1 × 10^–3^ Torr), and used as samples for the micro-PIXE camera. Each sample was irradiated with a 3 MeV proton beam (2 μm diameter), and the induced characteristic X-rays were detected using a Si (Li) detector. The signals were processed into images using Transform software (version 3.0; Fortner Software Inc. [13–15]). Nanoparticle rupture upon irradiation was categorized into three types: Type I, no observable morphological changes (Fig. 2D); Type II, release of the liquid core with an irregular nanoparticle contour (Fig. 2E); and Type III, release of the liquid core with no detectable nanoparticle contours (Fig. 2F). Type I nanoparticles were classified as unruptured, whereas Types II and III were identified as ruptured [11]. The rupture frequency was calculated as the mean percentage of ruptured particles to the total number of particles in 10 micro-PIXE views within a 25 × 25 μm scanning area. Eight mice were used.

### CT imaging of tumor and metastasis

After particle accumulation, CT (Micro CT LOCUS, CI 0014; GE Healthcare, Barrington, IL, USA) was performed under halothane anesthesia without fixation. The primary tumor and metastasis were detected, and the imaging ability of the nanoparticles was assessed by measuring the increased CT values of the primary tumor and the number of detectable metastases on CT. Eight mice were used.

### Irradiation

The mice were packed in a jig under normal breathing without anesthesia and the first radiation dose was administered. X-ray irradiation (10 or 20 Gy, 140 keV X-rays; Softex M150 WE, Softex, Tokyo, Japan) was delivered to the lungs and tumors at a dose rate of 0.315 Gy/min [9]. Total radiation doses were monitored using a radiation dose meter (RAMTEC; Toyo Medic Co., Ltd., Tokyo, Japan), and irradiation was automatically stopped upon reaching the target dose.

### Measurement of IFN-γ, perforin, and granzymes

IFN-γ, perforin, and granzymes were measured using the following ELISA kits: Mouse IFN-γ (RAB0225 Sigma Aldrich, St. Louis, MO, USA), Mouse Perforin I (PRF-1) (abx 154,527, Abexxa, Arlington, TX, USA), and Mouse Granzyme B (88–8022, Invitrogen, Waltham, MA, USA), respectively. The tumors were minced and digested in a buffer containing 2 mg/mL collagenase IV, 0.5 mg/mL hyaluronidase, and 0.1 mg/mL DNase I in DMEM. The resulting cell lysates were processed according to the manufacturer’s instructions, followed by a colorimetric assay at 450 nm. The quantification was based on a calibration curve. Eight mice were used in each experiment.

### Detection and assessment of HLA-A*02–01-GP-100 complex and CD^3+^ T-cells

Excised tumor and lung tissues preserved in Boulin’s fluid were routinely processed into 3-µm sections on silane-coated slides. After heat-mediated antigen retrieval with citrate buffer (pH 6), the samples were incubated with the primary antibody for GP-100 (PMEL/Melanoma gp100 (HMB45) Mouse mAb #38,815, Cell Signaling Technology, Danvers, MA, USA) at a 1/250 dilution, or anti-CD3^+^ antibody (Anti-Mouse CD3 antibody MAB4841-100, R&D Systems Inc., Minneapolis, MN, USA) at a 1:6400 dilution for 30 min at room temperature. A universal anti-mouse immunoperoxide polymer kit (HISTOFINE; Nichirei, Tokyo, Japan) was used for the secondary antibody. The chromogen was visualized using a DAB (3,3′-Diaminobenzidine tetrahydrochloride) and counterstained with hematoxylin. Induction of HLA-A*02–01-GP-100 was assessed by calculating the percentage of GP-100 positive cells across five fields at 400 × magnification. CD3^+^ T-cell infiltration was evaluated by counting the total number of CD3^+^ T cells in five fields under an optical microscope at 400 × magnification. Each experiment included eight mice.

### Antitumor effect and antimetastatic effects

In parallel with the experiments, eight to ten mice were subjected to the same two radiation sessions at doses of 10 or 20 Gy. The antitumor effects were assessed using a growth delay study. Tumor growth was monitored daily until the tumor diameter exceeded 12 mm. Absolute growth delay (AGD) was defined as the difference in the number of days required for treated and untreated tumors to grow from 8 to 12 mm in diameter. The normalized growth delay was calculated by subtracting the AGD of non-particle tebentafusp from the AGD of each treatment. The enhancement factors were calculated by dividing the normalized growth delay of each treatment by the AGD [16, 17]. The anti-metastatic effect was evaluated by counting the number of visible pulmonary nodules in the dissected lungs seven days after treatment.

### Statistical analysis

All statistical analyses were performed using analysis of variance (ANOVA). Data were tested for significance at P < 0.05. To allow for adequate ANOVA analysis, eight samples were used for each analysis.

## Results

### Generated nanoparticles before intravenous injection

The particle diameter measured under a phase-contrast microscope using the hemocytometer grid (50 µm), was 342 ± 56 nm (Fig. 2A). Micro-PIXE imaging of particles [13–15] detected the characteristic X-rays of Fe (Fig. 2C). The particle contours were smooth, and a high Fe content was observed (Fig. 2B).

### Morphology of injected particles, their kinetics in vivo and particle-assisted CT imaging

**Fig. 3:**
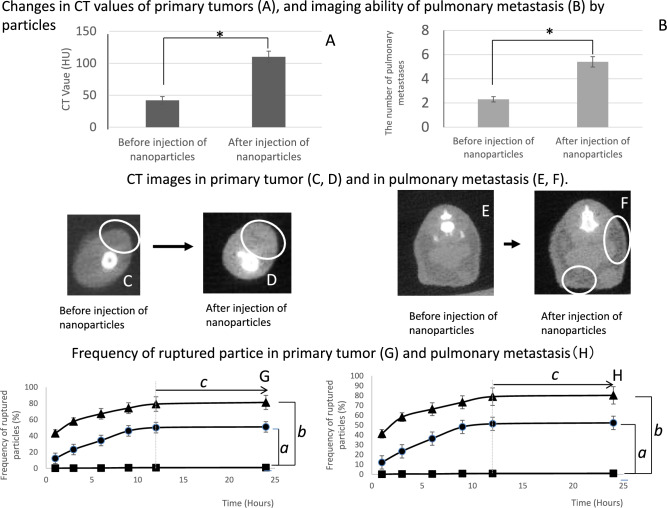
The power of imaging primary tumors and pulmonary metastasis, and drug delivery efficacy, using particle. **A**, **B**: Changes in CT values of primary tumors and imaging ability of pulmonary metastasis by particles. **A**: Alteration of CT-value by trapped particles. **B**: Alteration of detectable pulmonary metastasis. *Significant difference. **C**–**F**: CT images in primary tumor and in pulmonary metastasis. **C**: Primary tumor before intravenous injection of particles. **D**: Primary tumor after intravenous injection of particles. **E**: Pulmonary metastasis before intravenous injection of particles. **F**: Pulmonary metastasis after intravenous injection of particles.mG-H: Frequency of ruptured nanoparticles. **G**: primary tumor; **H**: pulmonary metastasis. *a*, *b* Significant differences. *c*: Completion of the rupturing process on 12 h after radiation. ■: No treatment, ●: 10-Gy irradiation, ▲: 20-Gy irradiation

IFN-γ-loaded particles at a concentration of 1 × 10^10^ were intravenously injected through the tail vein. Nanoparticles were observed in vivo using a micro-PIXE camera to distinguish them from blood cell components (Fig. 2D–F) [11, 13–15]. The IFN-γ particles transformed into a spindle shape after passing through the tumor capillaries (Fig. 2D) [11] and significantly increased in number 3 h post-injection (Fig. 2G, *). The accumulation was observed in primary tumors and surrounding muscle tissue (Fig. 2G, ●, ◆), as well as in pulmonary metastases and surrounding lung tissue (Fig. 2G, ■, ▲). Three hours after injection, the number of trapped IFNγ-particles in the primary tumor and pulmonary metastases (Fig. 2G, ●, ▲, *a*) was significantly higher than that in the surrounding muscles and lung tissue (Fig. 2G, ■, ◆*b*) (Fig. 2G, c). The IFN-γ-particles selectively accumulated in primary tumors and pulmonary metastases, with accumulations reaching completion at 9 h post-injection (Fig. 2G, ▲,●). CT imaging performed at this time showed a significantly higher in the CT-value of the primary tumor after IFN-γ particle injection than that before injection (Fig. 3A, *, C, D). The number of pulmonary metastases detected was higher than before the injection (Fig. 3B, E, F). These findings helped detect primary tumors and pulmonary metastases.

In order to study the safety profile associated with nanoparticles, the kinetics of particles in the liver (Fig. 2 H, ) and kidney (Fig. 2H, ) were also measured using a micro PIXE camera. No significant difference was observed in the number of particles between the kidney and liver (Fig. 2 H, , , *d*), and at each observation time point (Fig. 2H **). Their number was very small, at a maximum of 1.1 ± 0.09 particles within a scanning area of 25 × 25 µm, which was significantly lower than those in tumors and lung metastases (*cf* Fig. 2 G. *a*). Our particles were considered to have a sufficient safety profile.

### Administration of the first-radiation and radiation-induced rupturing of particles

After the CT scan, a first radiation dose of 10 or 20 Gy was administered. The particle rupture was tested using a micro-PIXE camera. No significant differences in radiation-induced IFN-γ particle rupture were observed between primary tumors and pulmonary metastases (Fig. 3G, H). The increase in ruptured IFN-γ particles in the irradiated group followed a radiation dose-dependent pattern, and the number of particles increased over time (Fig. 3G, H, ■, ●, ▲). A significant increase in rupturing was observed three hours after irradiation in the 10-Gy irradiation group (Fig. 3G, H, ●, *a*) and one hour after irradiation in the 20-Gy irradiation group (Fig. 3G, H, ▲, *b*). The process was almost completed 12 h after irradiation (Fig. 3G, H, ●, ▲, *c*), at which point released IFN-γ was detected and quantified.

## IFN-γ release from particles in primary tumor tissue and pulmonary metastases

**Fig. 4:**
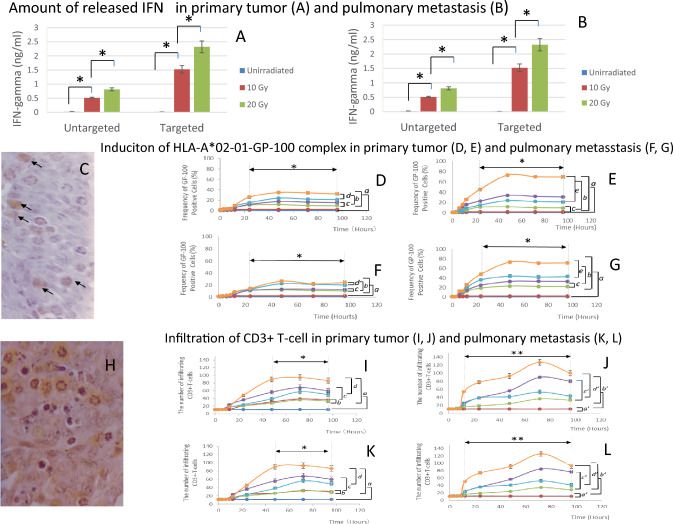
Releasing of IFN-γ from the particle, and IFNγ-mediated HLA-A*02–01-GP-100 complex and CD3^+^ T-cell infiltration. **A** and **B**: IFN-γ released from particles. **A**: Primary tumor, B: Pulmonary metastasis. *significantly different. : nonirradiated, : 10 Gy irradiated, : 20 Gy irradiated. **C**–**L**: Induction and kinetics of the HLA-A*02–01-GP-100 complex and infiltration of CD3^+^T-cells observed by immunohistochemical study.  No treatment; non-particlized or particlized IFN-γ only, :10-Gy irradiation only, :10-Gy irradiation with non-particlized or particlized IFN-γ, :20-Gy irradiation only, :20-Gy irradiation with non-particlized or particlized IFN-γ. **C**–**G**: Induction and kinetics of the HLA-A*02–01-GP-100 complex. **C**: Immunostained tissue images of HLA-A*02–01-GP-100 positive cells. The brown cells (arrows) were identified as HLA-A*02–01-GP-100 complex-positive cells. **D** and **E**: GP-100 expression in primary tumors. D: Non-particlized IFN-γ. **E**: Particlized IFN-γ. **F** and **G**: GP-100 expression in pulmonary metastases. **F**: Untargeted (non-particlized) IFN-γ. **G**: particlized (targeted) IFN-γ. *Significant increases in the induction of HLA-A*02–01-GP-100 complex from 24 to 96 h after irradiation, in the treatment groups receiving 10 or 20 Gy of radiation, compared with the non-irradiated treatment group. *a, b e:* significantly different. *c, d:* non-significant difference. **H**–**L**: Radiation recuruited infiltrattion of CD3^+^ T-cells. **H**: Immunostained CD3^+^ T-cells. The brown cells were identified as CD3^+^ T cells. **I** and **J**: Infiltrated CD3^+^ T cells in primary tumors. I: non-particlized IFN-γ. **J**: particlized IFN-γ. **K** and **L**: Infiltrated CD3^+^ T cells in pulmonary metastases. **K**: Non-particlized IFN-γ. **L**: Particlized IFN-γ. *Significant increases in the CD 3^+^ T-cell infiltration from 24 to 96 h after irradiation, in the treatment groups receiving 10 or 20 Gy of radiation, compared with the non-irradiated treatment group. **Significant increases in the CD ^3+^ T-cell infiltration from 48 to 96 h after irradiation, in the treatment groups receiving 10 or 20 Gy of radiation, relative to the non-irradiated treatment group, except for 20 Gy with particlized IFN-γ from 12 to 96 after treatment. *a, c, d:* Significantly different, *b:* Non-significantly different, *a’:* Non-significantly different, *b’, c’, d’:* Significantly different

No significant difference was observed in the amount of IFN-*γ* released between the primary tumorsand pulmonary metastases (Fig. 4A, , ,  to Fig. 4B , , , respectively). However, IFN-*γ* release significantly increased with radiation dose (Fig. 4A, B, *). Radiation targets IFN-γ during particle rupture.

### Induction of HLA-A*02–01-GP-100 complex by irradiation

HLA-A*02–01-GP-100 positive cells were detected as brown-colored cells by immunohistochemical staining (Fig. 4C, arrows). Significant increases in HLA-A*02–01-GP-100 positivity were observed in the treatment groups receiving 10 or 20 Gy radiation, starting 24 h after treatment and peaking at 48 h. The positivity rate remained high up to 96 h post-irradiation, in both primary tumors and pulmonary metastases (Fig. 4D, E, F, G , , , , *), when compared with unirradiated tumor groups (Fig. 4D, E, F, G, a) The induction of HLA-A*02–01-GP-100 increased in a radiation dose-dependent manner in both primary tumors (Fig. 4D, E, , ,*b*) and pulmonary metastases (Fig. 4F, G, , , *b*). No significant increase in HLA-A*02–01-GP-100 induction was observed with non-particlized IFN-γ combined with radiation (10-Gy: Fig. 4E, G, , , *c*; 20 Gy: Fig. 4E, G, , , *d*), in either primary tumors (Fig. 4D) or pulmonary metastases (Fig. 4F). When particlized IFN-γ were combined with radiation, no significant increase was observed with 10-Gy irradiation (Fig. 4D, F, , , *c*) but a significant increase was observed with 20-Gy irradiation (Fig. 4D, F, , , *e*), in both primary tumor (Fig. 4E) and pulmonary metastases (Fig.-4 G). The highest HLA-A*02–01-GP-100 induction was observed in the group that received 20 Gy radiation in combination with particlized IFN-γ in primary tumors (Fig. 4E, ).

### Infiltration of CD3^+^ T cells

**Fig. 5:**
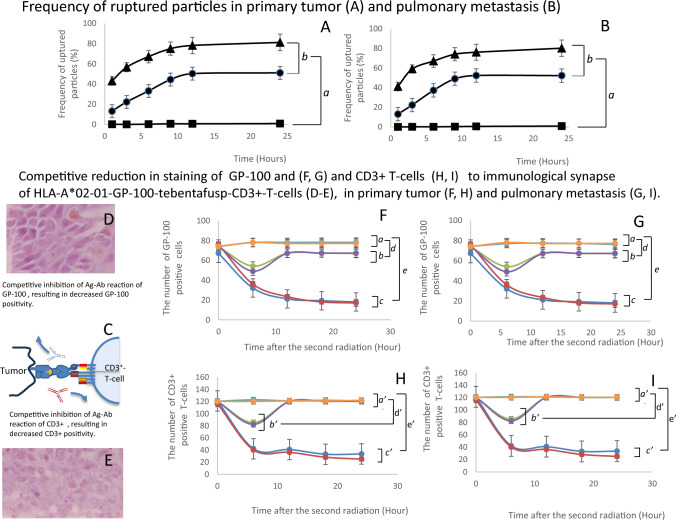
Release of tebentafusup in response to second radiation and subsequent formation of immunological synapse. **A**, **B**: The particle rupture caused by the second radiation exposure by micro-PIXE camera images. A: primary tumor. **B**: pulmonary metastasis. ■: No treatment ●: 10 Gy irradiation, ▲: 20 Gy irradiation. *a:* Significantly different from 3 to 24 h after radiation exposure. *b:* Significantly different from 1 to 24 h after radiation exposure. **C**–**I**: Immunological synapse formation of HLA-A*02–01-GP-100-tebentafusp-CD3^+^-T-cells, observed through competitive reduction in staining of CD3^+^ T-cells and GP-100. **C**–**D**: Competitive inhibition of GP-100 and CD3^+^ to immunological synapse, consisted of HLA-A*02–01-GP-100-tebentafusp-CD^3+^-T-cells. **D**: GP-100 microscopic images, **E**: CD3^+^ microscopic images. **I**: Immunological synapse formation observed through competitive inhibition. **F**, **H**: primary tumor; F: GP-100; **H**: CD3^+^ positivity. **G**, **I**: pulmonary metastasis. **F**: GP-100; **H**: CD3^+^ positivity. : 10 Gy only, : 20 Gy only, : 10 Gy plus non-particlized-tebentafusp, : 10 Gy plus unparticlized-tebentafusp : 10-Gy plus particlized tebentafusp, : 20-Gy plus particlized tebentafusp. *a, a’, b, b’, c, c’*: Non-significant different. *d, d’*: significantly different only on 6 h after second radiation. *e, e’*: significantly different in each observation time points

CD3^+^ T cells were detected as brown-colored cells by immunohistochemical staining (Fig. 4H). In the treatment group receiving radiation and/or non-particlized IFN-γ, each treatment (Fig. 4I, K, , , , , ) induced significantly greater CD3^+^ T-cell infiltration than the untreated group (Fig. 4I: primary tumor, K: pulmonary metastasis) starting at 36 h after treatment initiation (Fig. 4I, K, *, *a*).Non-particlized IFN-γ alone (Fig. 4I, K, ) and 10-Gy radiation alone (Fig. 4I, K, ) showed nearly equivalent ability to induce CD3^+^ T-cell infiltration (Fig. 4I, K, b). The combination of non-particlized IFN-γ with 10-Gy (Fig. 4I, K, ), or 20-Gy (Fig. 4I, K, ) of radiation significantly enhanced CD3^+^ T-cell accumulation compared with 10-Gy (Fig. 4I, K, ) or 20-Gy (Fig. 4I, K, ) of radiation alone, respectively (Fig. 4I, K 10-Gy; *c,* 20 Gy; *d*). In the treatment group receiving radiation and/or particlized IFNγ, administration of particlized IFNγ alone did not induce CD3^+^ T-cell accumulation (Fig. 4J: primary tumor, L: pulmonary metastasis , , *a’*). However, treatment-induced accumulation of CD3^+^ T-cells was observed only in the group treated (Fig. 4J, L, , , , , *b’*). The combination of particlized IFN-γ with 10-Gy (Fig. 4J, L, ) or 20-Gy radiation (Fig. 4J, L, ) induced significantly greater CD3^+^ T-cell infiltration than 10-Gy (Fig. 5C, E, ) or 20-Gy (Fig. 5C, E, ) radiation alone (Fig. 5C, E, 10-Gy; *c’,* 20 Gy; *d’*). Furthermore, the combination of particlized IFN-γ with 10-Gy (Fig. 4J, L , *c’*) or 20-Gy radiation (Fig. 4J, L , *c’*) induced significantly greater CD3^+^ T-cell infiltration than non-particlized IFN-γ with 10-Gy or (Fig. 4I, K, , *c’*) 20-Gy radiation (Fig. 4I, K, , *c’*). Significant increases in CD3^+^ T-cell infiltration were observed 24 h post-treatment (Fig. 4J, L, **), except in the 20-Gy radiation group, in which infiltration was observed by 12 h (Fig. 4J, L, ). The infiltration peaked at 72 h after treatment (Fig. 4J, L, , , , ), with significant increases persisting thereafter (Fig. 4J, L, **). The highest CD3^+^ T-cell infiltration occurred in the group treated with particlized IFN-γ and 20-Gy radiation, peaking 72 h post-radiation (Fig. 4J, L, ).

### Second radiation session

One hour after CT imaging, 1 × 10^10^ tebentafusp particles were injected intravenously through the tail vein. The second radiation was performed 72 h post-injection, after tebentafusp particle accumulation was completed under sufficient HLA-A*02–01-GP-100 complex induction and CD3^+^ T-cell infiltration. The following items were examined.

#### Rupturing of tebentafusp particles by second radiation

Measurements of tebentafusp particle rupturing caused by the second radiation were conducted similarly to those for IFN-γ particle rupture following the first irradiation (Fig. 5A, primary tumor; 5B, pulmonary metastasis). The kinetics of tebentafusp particle disruption following the second irradiation closely mirrored those observed for IFN-γ particles following the first irradiation (Fig. 5A, B, c, Fig. 3H). A significantly higher number of ruptured tebentafusp particles was observed in both 10-Gy (Fig. 5A, B, ●) and 20-Gy (Fig. 5A, B, ▲) irradiation groups than in non-irradiated groups (Fig. 5A, B, ■) three hours post-irradiation (Fig. 5A, B, *, *a*) in both the primary tumor (Fig. 5A) and pulmonary metastases (Fig. 5B). Moreover, 20-Gy irradiation induced (Fig. 5A, B, ●) significantly higher particle rupturing than 10-Gy irradiation (Fig. 5A, B, ▲) at one hour post-irradiation (Fig. 5A, B, b). The rupturing of tebentafusp particles followed a radiation dose-dependent pattern and was almost completed by 12 h post-irradiation (Fig. 5A, B, ●, ▲, *b*).

#### Immunological synapse formation

The formation of immunological synapses consisting of HLA-A*02–01-GP-100-tebentafusp-CD3^+^ T cells was evaluated by assessing competitive inhibition of binding between GP-100 and CD3^+^ antibodies by tebentafusp (Fig. 5C–E). No competitive inhibition was observed in the group receiving 10 Gy or 20 Gy of the second radiation with particles without tebentafusp (Fig. 5F–I, , , *a, a’*). Competitive inhibition was observed in groups that were administered non-particlized or particlized tebentafusp in combination with a second radiation dose. (Fig. 5F–I, , , , , *b, b’, c, c’*).

In the non-particlized tebentafusp group, transient decreases were observed in both CD3^+^ T cells and HLA-A*02–01-GP-100-positive cells until 6 h after the second radiation in the primary tumor (Fig. 5F: GP-100, H: CD3^+^ T cell, : 10-Gy : 20 Gy,* b, d*) and pulmonary metastasis (Fig. 5G: GP-100, I: CD3^+^ T cell, : 10-Gy : 20 Gy, *b’, d’*). Subsequently, both cell counts returned to pretreatment levels (Fig. 5F, I, , ,*b, b’ d, d’*).

In contrast, in the group receiving particlized tebentafusp, the number of CD3^+^ T cells and HLA-A*02–01-GP-100-positive cells steadily decreased and was completed by 18 h after the second radiation in the primary tumor (Fig. 5F: GP-100, H: CD3^+^ T-cell, : 10-Gy, : 20 Gy*, c, e*) and pulmonary metastasis (Fig. 5G: GP-100, I: CD3^+^ T-cell, : 10-Gy, : 20 Gy, *c’,e’*).

These results suggest that in the non-particlized tebentafusp group, the immunological synapse consisting of HLA-A*02–01-GP-100-tebentafusp-CD3^+^ T cells persisted for 6 h after the second radiation but subsequently disappeared. Conversely, in the particlized tebentafusp group, the immunological synapses were sustained beyond 6 h post-irradiation. However, no significant differences were observed in the formation of HLA-A*02–01-GP-100-tebentafusp-CD3^+^ T-cell complexes between the 10-Gy and 20-Gy second radiation groups.

#### Release of granzymes and perforin from CD3^+^ T cells

**Fig. 6:**
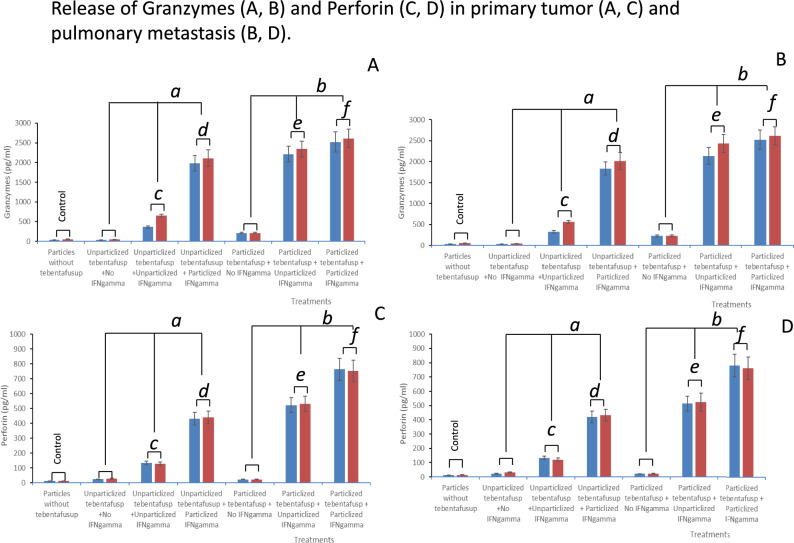
Release of Peferforin/Granzymes. **A**: granzymes in primary tumor, **B**: granzymes in pulmonary metastasis, **C**: perforin in primary tumor, and **D**: perforin in pulmonary metastasis. : 10 Gy, : 20 Gy, *a, b:* significantly different. *c, d, e, f:* Non-significantly different. The amount of Granzymes/Perforin, released by treatment with not containing tebentafusp, was shown as a “control” for comparison

The released granzymes and perforin were measured 6 h after the second radiation dose, coinciding with the observed immunological synapse formation in each treatment group receiving non-particlized or particlized tebentafusp with a second irradiation of 10 or 20 Gy in both the primary tumor and pulmonary metastasis (Fig. 6A: granzymes in primary tumor, B: granzymes in pulmonary metastasis, C: perforin in primary tumor, and D: perforin in pulmonary metastasis).

Granzymes and perforin exhibited similar release kinetics in primary tumors and pulmonary metastases (Fig. 6A–D). This release was observed only in groups that received non-particlized or particlized IFN-γ during the first radiation session (Fig. 6A–D, a, b). Particlized IFN-γ enhanced the release of granzyme and perforin compared with non-particlized IFN-γ (Fig. 6A–D, comparisons *c/e* to *d/f*, respectively). Similarly, particlized tebentafusp released more granzyme/perforin than non-particlized tebentafusp (Fig. 6A–D, comparisons *c/d* to *e/f*, respectively); however, no significant difference was observed in granzyme or perforin release between the 10-Gy and 20-Gy second radiation doses when combined with either particlized or non-particlized-tebentafusp plus particlized or non-particlized IFN-γ (Fig. 6A–D, c, d, e, f).

The highest levels of granzymes and perforin release were observed in the combination treatment group of particlized tebentafusp + particlized IFN-γ + 10 Gy or 20 Gy second irradiation (Fig. 6f). The levels were not significantly different between the groups.

### Antitumor and antimetastatic effect

#### Antitumor effect

**Fig. 7:**
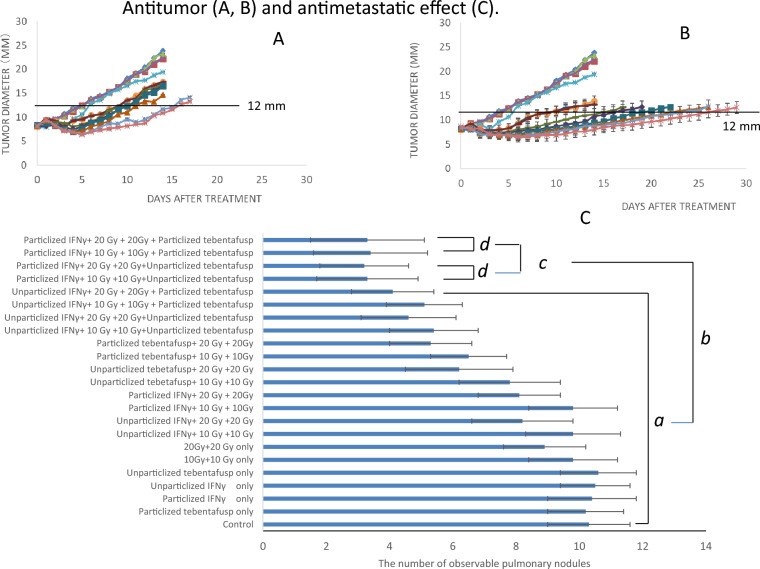
Antirumor and Antimetastatic effect. **A**, **B**: Antitumor effect: tumor diameter versus time (days). **A**: 10 Gy, B: 20 Gy, : No treatment, : Particlized tebentafusp only, : Particlized IFN-γ only. : Non-particlized IFN-γ only, Non-particlized tebentafusp only, : Radiation only, **:** Non-particlized IFN-γ plus radiation,: Particlized IFN-γ plus radiation.: Non-particlized tebentafusp plus radiation, : Particlized tebentafusp plus radiation. : Non-particlized IFN-γ plus radiation plus non-particlized tebentafusp. : Non-particlized IFN-γ plus radiation plus particlized tebentafusp.  ◆:Particlized IFN-γ plus radiation plus non-particlized tebentafusp. Particlized IFN-γ plus radiation plus particlized tebentafusp. **C**: Antimetastatic effects by counting metastatic pulmonary nodules *a:* No significantly different; *b:* Significantly different *c, d:* No significantly different

Tumor growth was measured as the time required for the tumor to grow from 8 to 12 mm (Fig. 7A, B), and growth delay metrics (absolute, normalized, and enhancement factors) were calculated (Table 1, *a-p*).

**Table 1:**
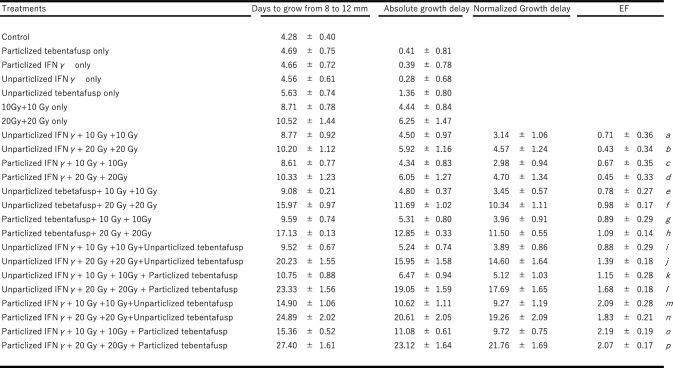
Antitumor effect by tumor growth delay assay

No significant combination effect was observed with particlized or non-particlized tebentafusp combined with either first-10-Gy plus second-10-Gy radiation (Fig. 7A, ◆, **-**, Table 1*e, g*,) or first-20-Gy plus second-20-Gy radiation (Fig. 7B, ◆, **-**, Table 1*f, h*). Similarly, no effect was observed with non-particlized IFN-γ, non-particlized tebentafusp, and first-10-Gy plus second-10-Gy radiation (Fig. 7A, +, Table 1 i). However, combining non-particlized IFN-γ or non-particlized tebentafusp with first-20-Gy plus second-20-Gy radiation showed a combination effect with an enhancement factor (EF) of 1.39 (Fig. 7B, +**,** Table 1*j*).

Under particlized IFN-γ, a significant antitumor effect was observed when non-particlized and particlized tebentafusp were combined with radiation (Fig. 7A, B, , , Table-1, *m, n. o, p*). Notable combined antitumor effects were observed with particlized IFN-γ + first-10-Gy + second-10-Gy + particlized tebentafusp (EF 2.19 ± 0.19, Fig. 7A, , Table 1*o*) and particlized IFN-γ + first-20-Gy + second-20-Gy + particlized tebentafusp (EF 2.07 ± 0.17, Fig. 7B, , Table 1*p*). No significant difference was observed in the antitumor effect between the first-10-Gy + second-10-Gy and first-20-Gy + second-20-Gy irradiation (Fig. 7A, B, , Table 1. *o, p*) in the particlized IFN-γ + particlized tebentafusp + radiation combination group. Similarly, no significant difference was observed in the release of perforin/granzyme between the 10-Gy and 20-Gy second irradiations in this combination group (Fig. 6, *d*).

#### Antimetastatic effect

The number of observable pulmonary nodules seven days after treatment initiation is shown in Fig. 7C. Fewer metastases were observed with the combination of non-particlized tebentafusp + first-20-Gy + second-20-Gy. Metastases were also reduced with a combination of particlized tebentafusp + radiation (first-10-Gy + second-10-Gy or first-20-Gy + second-20-Gy), non-particlized IFN-γ + radiation (first-10-Gy + second-10-Gy or first-20-Gy + second-20-Gy) + non-particlized tebentafusp, and non-particlized IFN-γ + radiation (first-10-Gy + second-10-Gy or first-20-Gy + second-20-Gy) + particlized tebentafusp. However, these reductions were not significant compared with the control (no treatment) (Fig. 7C a). A significant reduction in lung metastases was observed in the groups receiving particlized IFN-γ and radiation (first-10-Gy + second-10-Gy, or first-20-Gy + second-20-Gy), with or without non-particlized or particlized tebentafusp (Fig. 7C, b). No significant changes were observed with radiation dose or tebentafusp particlization (Fig. 7C, c, d.)

## Discussion

Theranostics has been implemented primarily in the field of nuclear medicine, which requires limited availability of nuclear medicine imaging equipment and nuclear medicine treatment isolation wards [18]. In this study, we investigated theranostics using CT and CT-detectable nanoparticles that release their contents upon radiation [9–11]. Because CT is widely available and the nanoparticles do not require radioisotope treatment wards, which extend theranostics from medical institutions with nuclear medicine facilities to medical institutions without nuclear medicine facilities.

We previously proposed theranostic nanomedicine [8–10, 19–21] using CT-detectable nanoparticles that released IFN-γ or tebentafusp after two radiation sessions. We investigated two key issues: (1) particle imaging using CT and (2) the synergistic effect of radiation and immunotherapy.

### Tumors and metastasis imaging by CT and safety profile of our particles

The particles formed through the Fe polymerization of hyaluronate and alginate were detectable on CT. They accumulated in the primary tumors and pulmonary metastases by leaking through the coarse endothelium of the tumor vasculature (Fig. 2G, a) [11, 12], increasing primary tumor density (Fig. 3A, C, D) and the number of detectable pulmonary metastases (Fig. 3B, E, F), resulting in clearer imaging of these malignant lesions. And our particles accumulated in primary tumors and pulmonary metastases, but not in normal tissue around the tumor, normal lung tissue, kidneys, or liver (Fig. 2G *b*, H *d*). This suggests that the safety of drugs, released from the particles, may be improved and side effects reduced. Further research is needed regarding the safety of the particles and reducing the side effects of the released drugs.

### Synergistic effects of two radiation sessions and immunotherapy with particlized tebentafusp

During the first radiation session, IFN-γ nanoparticles released IFN-γ (Fig. 4A, B, *). The released IFN-γ enhanced HLA-A*02:01-GP-100 induction by radiation (Fig. 4C–G, a) [4–6], and cooperated with radiation to recruit CD3^+^ T cells (Fig. 4H–L, a) [1, 5, 6]. After the second irradiation, the particles released tebentafusp in a dose-dependent manner. (Fig. 5A, B). Released tebentafusp formed HLA-A*02–01-GP-100-tebentafusp-CD3^+^ T-cell complexes, enhanced by the particlization of IFN-γ and tebentafusp due to continuous drug release from irradiated particles (Fig. 5D–G, : 10-Gy, : 20-Gy) [9–11]. The two radiation sessions significantly increased the antitumor effects on primary tumors and pulmonary metastases. However, increasing the radiation dose from first-10-Gy/second-10-Gy to first-20-Gy/second-20-Gy did not enhance immunological synapse formation (Fig. 5D–G, , :), increase granzyme/perforin release (Fig. 6A-D, c, d, e, f), or improve antitumor activity (Fig. 7A, B, , Table-1. *o, p*) or antimetastatic effects (Fig. 7C, c, d). A possible explanation for this phenomenon is FAS-L-mediated CD3^+^ T-cell toxicity, which suppresses CD3^+^ T cells and induces apoptosis [22–24]. Research on FAS-L is currently underway [25–27].

## Conclusions

1. CT-imageable particles accumulated in the primary tumors and pulmonary metastases by leaking through the coarse endothelium of the tumor vasculature, resulting in clearer imaging of these malignant lesions.
2. The accumulated particles released IFN-γ and tebetafusup upon first and second radiation, respectively. Released IFN-γ recruited the CD3^+^-T-cells to the irradiated field. And Released tebentafusp formed HLA-A*02–01-GP-100-tebentafusp-CD3^+^ T-cell complexes, enhanced by the particlization of IFN-γ and tebentafusp due to continuous drug release from irradiated particles.
3. Those 1 and 2 described above allowed us theranostic nanomedicine.
4. In order to enhance the antitumor and antimetastatic effect, researches for FAS-L-mediated CD3^+^ T-cell toxicity is needed. Regarding the safety of the particles and reducing the side effects of the released drugs, further research is needed.

## References

[1] Guo S, Yao Y, Tang Y, Xin Z, Dang Wu, Ni C, et al. Radiation-induced tumor immune microenvironments and potential targets for combination therapy. Signal Transduct Target Ther. 2023;8:205–26. 10.1038/s41392-023-01462-z. 37208386 10.1038/s41392-023-01462-z PMC10199044

[2] Gallegos CE, Michelin S, Duvner D, Arosella ED. Immunomodulation of classical and non-classical HLA modulation by ionizing radiation. Cell Immunol. 2016;303:16–23. 10.1016/j-cellimm.2016.04.005. 27113815 10.1016/j.cellimm.2016.04.005

[3] Uchihara Y, Bunga T, Permata M, Sato H, Kawabata-Iwakawa R, Katada S, Wenchao Gu, et al. DNA damage promotes HLA classI presentation by stimulating a pioneer round of translation-associated antigen production. Mol Cell. 2022. 10.1016/j.molcel.2022.04.030. 35594857 10.1016/j.molcel.2022.04.030

[4] Martinez-Perez D, Vinal D, Solares I, Espinosa E, Feliu J. GP-100 as a Novel therapeutic target in uveal melanoma. Cancer. 2021;13:5968–77. 10.3390/cancers13235968. 10.3390/cancers13235968 PMC8656894 34885078

[5] Jorgovanovic D, Song M, Wang L, Zhang Yi. Roles of IFN-γ in tumor progression and regression. Biomarker Research. 2020;8:1–16. 10.1186/s40364-020-00228-x. 33005420 10.1186/s40364-020-00228-x PMC7526126

[6] Li Ji, Ybarra R, Mak J, Herault A, De Almeida P, Arrazate A, Ziai J, Totpal K, Junttila MR, Walsh KB. IFNγ-induced chemokines are required for CXCR3-mediated T-cell recruitment and antitumor efficacy of anti-HER2/CD3Bispecific antibody. Clin Cancer Res. 2018;24(24):6447–58. 10.1158/1078-0432.CCR-18-1139. 29950350 10.1158/1078-0432.CCR-18-1139

[7] Middleton MR, McAlpine C, Woodcock VK, Corrie P, Infante JR, Steven NM, et al. Tabentafusp, A TCR/Anti-CD3 bispecific fusion protein targeting gp100, potently activated antitumor immune responses in patients with metastatic melanoma. Clin Cancer Res. 2020;26:5869–78. 10.1158/1078-0432.CCR-20-1247. 32816891 10.1158/1078-0432.CCR-20-1247 PMC9210997

[8] Hassel JC, Berking C, Forschner A, Gebhardt C, Heinzerling L, Meier F, et al. Practical guidelines for the management of adverse events of the T cell engager bispecific tebentafusp. Eur J Cancer. 2023;191:112986–98. 10.1016/j.ejca.2023.112986. 37595494 10.1016/j.ejca.2023.112986

[9] Harada S, Ehara S, Ishii K, Hiromichi Yamazaki Y, Shigeo Matsuyama Y, Sato T, et al. Targeted delivery of chemotherapeutic agents using improved radiosensitive liquid core microcapsules and assessment of their antitumor effect. Int J Radiation Oncol, Biol, Phys. 2009;75(2):455–62. 10.1016/j.ijrobp.2009.02.082. 10.1016/j.ijrobp.2009.02.082 19735868

[10] Harada S, Ehara S, Ishii K, Sato T, Koka M, Kamiya T, et al. Targeted concurrent chemoradiotherapy, by using improved microcapsules that release carboplatin in response to radiation, improves detectability by computed tomography as well as antitumor activity while reducing adverse effect in vivo. Biomed Pharmacother. 2015;70:196–205. 10.1016/j.biopha.2015.01.006. 25776501 10.1016/j.biopha.2015.01.006

[11] Segawa T, Harada S, Sato T, Ehara S. Delivery and effectiveness of carboplatin via targeted delivery compared to passive accumulation of intravenously injected particles releasing carboplatin upon irradiation. Radiation Res. 2020;193:263–73. 10.1667/RR15357.1. 31910093 10.1667/RR15357.1

[12] Bertrand N, Wu J, Xu X, Kamaly N, Farokhzad OC. Cancer nanotechnology: the impact of passive and active targeting in the era of modern cancer biology. Adv Drug Deliv Rev. 2014;66:2–25. 10.1016/j.addr.2013.11.009. 24270007 10.1016/j.addr.2013.11.009 PMC4219254

[13] Matsuyama S, Ishii K, Sugimoto A, Satoh T, Gotoh K, Yamazaki H, et al. Development of Micro-PIXE camera. Int J PIXE. 1998;8:203–8. 10.1142/S0129083502000160.

[14] Sugimoto A, Ishii K, Matsuyama S, Satoh T, Gotoh K, Yamazaki H, et al. Application of Micro-PIXE camera to elemental analysis of a single cell. Int J PIXE. 1999;9:151–60. 10.1142/S0129083502000160.

[15] Campbell JL, Russell SB, Faiq S, Schulte CW, Ollerhead RW, Gingerich RR. Optimization of PIXE sensitivity for biomedical applications. Nucl Inst Meth. 1981;181:285–92. 10.1289/ehp.93101302.

[16] Milas L, Mason K, Hunter N, Petersen S, Yamakawa M, Ang K, et al. In vivo enhancement of tumor radioresponse by C225 antiepidermal growth factor receptor antibody. Clin Cancer Res. 2000;6:701–8. 10690556

[17] Milross CG, Mason KA, Hunter NR, Terry NH, Patel N, Harada S, et al. Enhanced radioresponse of paclitaxel-sensitive and -resistant tumours in vivo. Eur J Cancer. 1997;33:1299–308. 10.1002/ijc.10907. 9301459 10.1016/s0959-8049(97)00107-x

[18] Marini JFG, Nunes RF, Coutinho AM, Zaniboni EC, Costa LB, Barbosa FG, Queiroz MA, Cerri GG, Cuchipigue CA. Theranostics in nuclear medicine: emerging and reemerging integrated imaging and therapies in the era of precision oncology. Radiographics. 2020. 10.1148/rg.2020200021. 10.1148/rg.2020200021 33001789

[19] Strebhardt K, Ullrich A. Paul Ehrlich’s magic bullet concept: 100 years of progress. Nat Rev Cancer. 2008;8(6):473–80. 10.1038/nrc2394. 18469827 10.1038/nrc2394

[20] Hricak H. Oncologic imaging: a guiding hand of personalized cancer care. Radiology. 2011;259(3):633–40. 10.1148/radiol.11110252. 21493796 10.1148/radiol.11110252

[21] Jadvar H, Chen X, Cai W, Mahmood U. Radiotheranostics in cancer diagnosis and management. Radiology. 2018;286(2):388–400. 10.1148/radiol.2017170346. 29356634 10.1148/radiol.2017170346 PMC5790308

[22] Mazar J, Thomas M, Bezrukov L, Chanturia A, Pekkurnaz G, Yin S, Kuznetsov SA, Robey PG, Zimmerberg J. Cytotoxicity mediated by the Fas Ligand (FasL)-activated apoptotic pathway in stem cells*. J Biol Chem. 2009;284(33):22022–8. 10.1074/jbc.M109.032235. 19531476 10.1074/jbc.M109.032235 PMC2755926

[23] Akashi A, Kuwano K. Fas-mediated lysis of target cell by IL-2 treated cytotoxic T lymphocytes. Kurume Med J. 2002;49:119–29. 12471726 10.2739/kurumemedj.49.119

[24] Ni X, Chunle IZ, Talpur R, Duvic M. Resistance to activation-induced cell death and bystander cytotoxicity via the Fas/Fas ligand pathwayare implicated in the pathogenesis of cutaneous Tcell lymphomas. J Invest Dermatol. 2005;124(4):741–50. 10.1111/j.0022-202X.2005.23657.x. 15816832 10.1111/j.0022-202X.2005.23657.x

[25] Singh R, Kim Y-H, Lee S-J, Eom H-S, et al. 4–1BB immunotherapy: advances and hudles. Exp Mole Med. 2024;56:32–9. 10.1038/s12276-023-01136-4. 10.1038/s12276-023-01136-4 PMC10834507 38172595

[26] Kristin GA, Shannon KO, Breanna MB, et al. Engineering adoptive T cell therapy to co-opt FAS ligand-mediated death signaling in ovarian cancer enhances therapeutic efficacy. J Immunother Cancer. 2022;10:e003959. 10.1136/jitc-2021-003959. 35264436 10.1136/jitc-2021-003959 PMC8915280

[27] Oda SK, Anderson KG, Ravikumar P, et al. A-Fas-4–1BB fusion protein converts a death to a pro-survival signal and enhances T cell therapy. J Exp Med. 2020;217(12):e20191166. 10.1084/jem.20191166. 32860705 10.1084/jem.20191166 PMC7953733

